# Massive pulmonary embolism led to cardiac arrest two days after thoracoscopy in a young male with pleural tuberculosis

**DOI:** 10.1002/rcr2.1069

**Published:** 2023-05-08

**Authors:** Wael Kanjo, Shahem Abbarh, Amina Bougaila, Nagham Sadik, Mhd Baraa Habib

**Affiliations:** ^1^ Department of Internal Medicine Hamad Medical Corporation Doha Qatar

**Keywords:** cardiac arrest, pulmonary embolism, thoracoscopy, tuberculosis, venous thromboembolism

## Abstract

TB itself is considered an independent risk factor for VTE; however, developing pulmonary embolism after medical thoracoscopy is extremely rare. Herein, we describe a 30‐year‐old previously healthy male with pleural tuberculosis developed a massive pulmonary embolism with subsequent cardiac arrest after a diagnostic medical thoracoscopy. Computed tomography pulmonary angiogram (CTPA) showed major right pulmonary embolism (PE). Unfortunately, the patient passed away despite resuscitation and extensive organ support in the intensive care unit (ICU). This case highlights the thrombotic risk in this population group in order to avoid such devastating complications.

## Graphical abstract

We describe a fatal case of a 30‐year‐old previously healthy male with pleural tuberculosis who developed a massive pulmonary embolism with subsequent cardiac arrest after a diagnostic medical thoracoscopy. Computed tomography pulmonary angiogram showed major right pulmonary embolism. Unfortunately, the patient passed away despite resuscitation and extensive organ support in the intensive care unit. This case highlights the thrombotic risk in this population group in order to avoid such devastating complications.

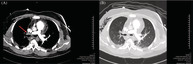

## INTRODUCTION

Tuberculosis (TB), caused by a microorganism called *Mycobacterium tuberculosis*, is a major contagious disease and health threat worldwide. In 2020, TB was the second leading infectious cause of death worldwide after coronavirus disease 2019 (COVID‐19), according to the World Health Organization (WHO).^1^ TB may involve different organs leading to diverse symptoms and signs. The lungs are affected in most cases (approximately 85%); however, any extrapulmonary site can be involved.^2^ Extrapulmonary TB includes, but is not limited to, TB lymphadenitis, pleural TB, and abdominal TB. In addition, TB, whether pulmonary or extrapulmonary, has been considered an independent risk factor for a hypercoagulable state predisposing to venous thromboembolism (VTE).^3^ Furthermore, VTE can occur in the early or late stages of the clinical TB course and rarely happens in asymptomatic TB patients.^4^

Pleural TB is one of the most common forms of extrapulmonary TB and is a significant cause of pleural effusion in endemic tuberculosis areas.^5^ A definitive diagnosis of tuberculous pleural effusion can be established through pleural fluid analysis or a pleural biopsy specimen, which can be obtained via closed percutaneous needle biopsy or thoracoscopy (medical or video‐assisted thoracoscopy surgery “VATS”).^6^ Medical thoracoscopy (MT) is a minimally invasive procedure that examines the pleural surface and allows several pleural procedures, including biopsy.^7^ When performed by experienced physicians, MT is generally a safe procedure. Reported complications of medical thoracoscopy include lung laceration, bleeding, prolonged air leak, subcutaneous emphysema, and mortality.^7^, ^8^ Venous thromboembolism (VTE) has rarely been reported as a complication following MT.^9^ Herein, we present a case of a previously healthy young man who was diagnosed with pleural TB based on a pleural biopsy via MT. Shortly after the procedure, he developed a massive pulmonary embolism and cardiac arrest.

## CASE REPORT

A 30‐year‐old Indian non‐smoker gentleman with no significant previous medical history presented to the emergency department with a one‐week history of subjective fever, dry cough, and mild exertional dyspnea. He denied any chest pain or hemoptysis. There was no sick contact, but he had a recent travel to India 6 months prior to the presentation. On presentation, he had tachycardia of 130 beats/minute, fever of 38 degrees Celsius, respiratory rate of 22, oxygen saturation of 99% on room air, and blood pressure was 147/80 mmHg. Chest examination showed decreased air entry and decreased tactile vocal fremitus in the right middle and lower zones, with dullness to percussion and no wheezes or crackles. Other physical examinations were unremarkable.

**FIGURE 1:**
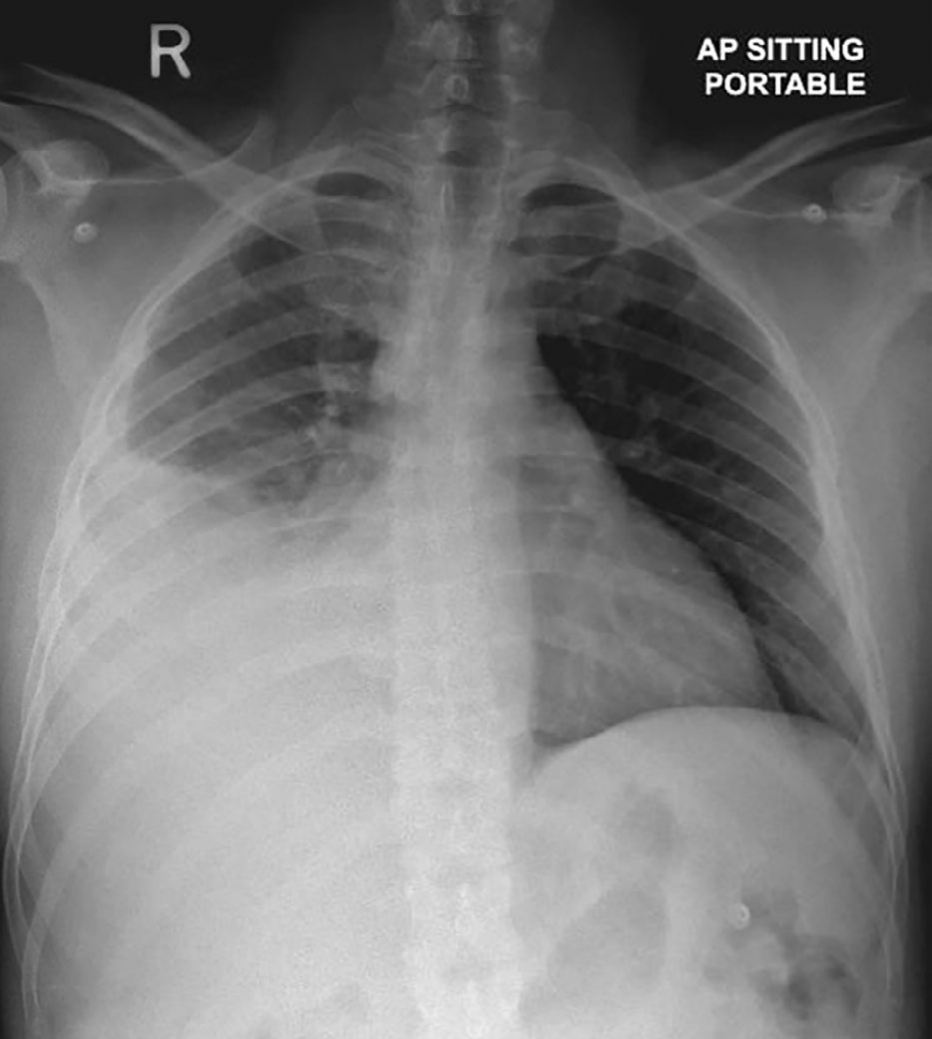
Chest X‐ray showing moderate to large amount of right‐side pleural effusion with adjacent atelectasis/consolidation

**FIGURE 2:**
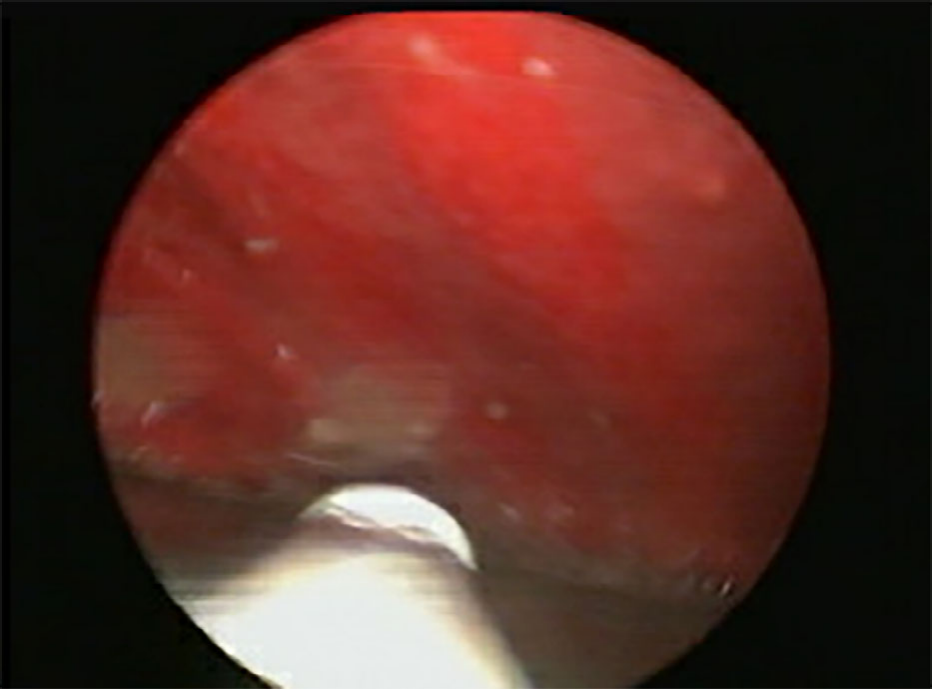
Inflamed parietal pleura with sago like whitish nodules suggestive of pleural tuberculosis

Electrocardiogram (ECG) showed sinus tachycardia. Laboratory investigations revealed elevated C‐reactive protein (CRP) 176 mg/L (0–5 mg/L) and positive interferon‐gamma release assays (IGRA), but otherwise unremarkable. Chest X‐ray showed moderate to large right‐side pleural effusion with adjacent atelectasis/consolidation (Figure 1). The patient was initially started on ceftriaxone and azithromycin for probable community‐acquired pneumonia. Pleural fluid analysis, obtained through diagnostic thoracentesis, revealed exudative effusion with a lymphocytic predominance (Table 1). Sputum and pleural fluid samples were negative for acid‐fast bacilli (AFB) smear and TB polymerase chain reaction (PCR). Computed tomography (CT scan) of the thorax showed large right‐side pleural effusion with underlying lower lobe collapse and atelectasis, as well as upper abdominal and left axillary lymphadenopathy of low attenuation concerning for necrotic changes. MT, done on the eighth day of admission to obtain a pleural biopsy, showed inflamed pleura with whitish nodules suggestive of TB (Figure 2). Therefore, the patient was started on an anti‐TB regimen. The pleural biopsy later revealed necrotizing granulomatous inflammation, with a negative AFB smear and a positive *M. tuberculosis* culture.

**TABLE 1:** Pleural fluid analysis

Test	Result
Colour	Turbid
Glucose	5.3 mmol/L
LDH	680 U/L
pH	7.59
Protein	61 g/L
Albumin	27 g/L
WBC	5100/μL
RBC	7600/μL
Neutrophils	30%
Lymphocytes	60%
Quantiferon TB Gold Plus	Positive

Abbreviations: LDH, lactate dehydrogenase; RBC, red blood cells; TB, tuberculosis; WBC, white blood cells.

**FIGURE 3:**
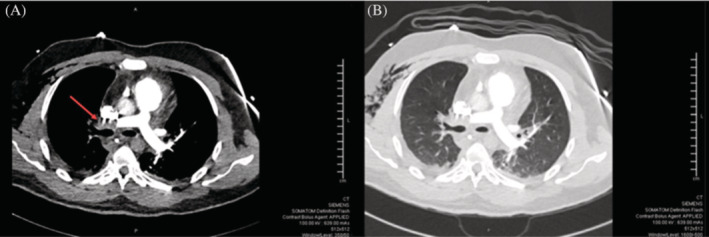
Computed tomography pulmonary angiogram (CTPA) with mediastinal (A) and lung (B) windows showing major right artery pulmonary embolism with reduced vascularity of the right lung

**FIGURE 4:**
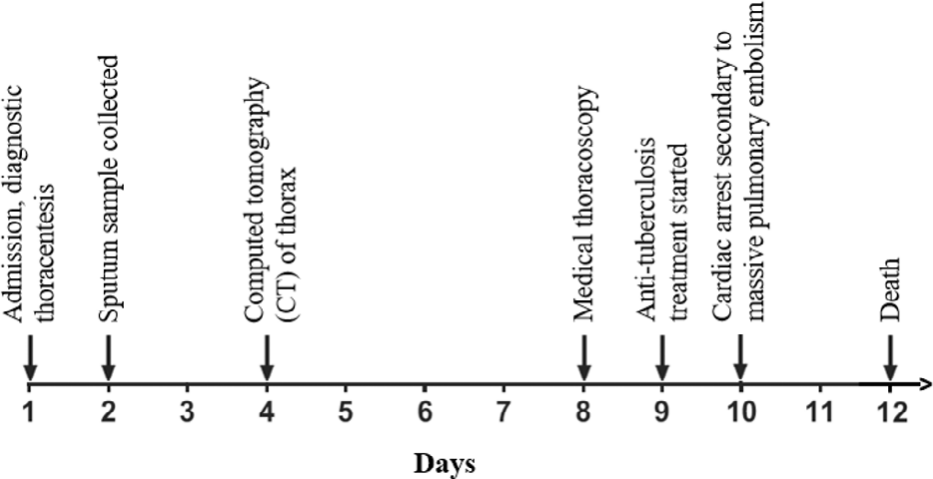
Timeline of important clinical events during the hospital stay

After the thoracoscopy, the patient was vitally stable and clinically doing well, apart from mild chest pain at the site of the procedure. However, 2 days later, he developed sudden respiratory distress, tachycardia, and tachypnea with a drop of oxygen saturation to 88% on room air. The cardiopulmonary examination was only significant for tachycardia. Arterial blood gas (ABG) showed hypoxemia with respiratory and lactic acidosis. The patient soon became gasping, then unresponsive and pulseless. Cardiopulmonary resuscitation (CPR) was immediately initiated, the patient was intubated, and extracorporeal membrane oxygenation (ECMO) was connected. Urgent CT pulmonary angiogram (CTPA) showed complete occlusion of the right main pulmonary artery with reduced vascularity of the right lung (Figure 3). It is worth mentioning that the patient was receiving VTE prophylaxis with enoxaparin 40 mg subcutaneously daily during the hospital course, but it was suspended 1 day before the procedure. The patient was kept in the intensive care unit (ICU). He developed anuric acute kidney injury, requiring haemodialysis, and a shock liver with severe coagulopathy. After 2 days in the ICU, he developed pulseless ventricular tachycardia and passed away despite resuscitation. A timeline of the clinical events during the patient's hospital stay is depicted in Figure 4.

## DISCUSSION

TB is an ancient and tricky infectious disease caused by *M. tuberculosis*.^10^ TB can cause various symptoms and signs depending on the infected organs. In most cases, TB involves the lungs, where it can cause lung damage by a variety of pulmonary pathologies, including cavitation, fibrosis, or nodular infiltrates.^10^ Less commonly, in about 15% of cases, TB may involve organs other than lungs (i.e., extrapulmonary) such as lymph nodes, pleura, gastrointestinal tract, and central nervous system.^2^

Regardless of the involvement, TB has been found to be associated with a hypercoagulable state that may increase the risk for VTE.^3^, ^11^ In a recent systematic review and meta‐analysis involving more than 16,000 active TB patients, the prevalence of VTE events was 3.5%. This was divided further into pulmonary embolism (PE) and deep venous thrombosis (DVT) components, with a prevalence of 5.8% and 1.3%, respectively.^12^ The pathogenesis behind the increased risk of VTE in patients with TB is not fully understood; however, alterations in the three components of Virchow's triad are thought to be at the core of the pathophysiology.^13^, ^14^

The broad spectrum of disease presentation and multisystem involvement may sometimes cause a diagnostic dilemma for the treating physician. The diagnosis of tuberculosis should be suspected in patients with relevant clinical manifestations and epidemiologic factors.^15^ Definitive diagnosis, however, is established by isolation of *M. tuberculosis* from a bodily secretion or fluid (e.g., a culture of sputum, bronchoalveolar lavage, or pleural fluid) or tissue (e.g., pleural biopsy or lung biopsy).^15^ Our patient presented with a cough and fever and was found to have moderate to large right‐side pleural effusion. In the context of his clinic presentation and a history of recent travel to a TB endemic country, pleural TB was highly suspected. His pleural fluid analysis revealed exudative effusion with a lymphocytic predominance; however, AFB smear and TB PCR in the pleural fluid were negative. Therefore, MT was done to obtain a pleural biopsy in order to establish a definitive diagnosis.

MT allows for direct visualization of the pleura. Its application is mainly for diagnosing pleural effusion and performing talc poudrage pleurodesis.^16^ Overall, MT is a well‐tolerated and safe procedure with a low complication rate.^16^ Complications of MT may include major complications such as lung laceration, pulmonary re‐expansion edema, air embolism, and rarely death. At the same time, minor complications include pain, cutaneous infection at the entry site, fever, and subcutaneous emphysema.^8^, ^17^ In a large retrospective study that included 1926 patients who underwent MT (either diagnostic or therapeutic), mortality was reported in only one patient (0.1%) who was in the therapeutic MT group.^7^ In the diagnostic MT group (*n* = 662), pain followed by subcutaneous emphysema was the most commonly reported complication, with no described VTE events. VTE complications following medical thoracoscopy are rarely seen. Rodríguez‐Panadero reported a 2.7% prevalence of PE in 411 patients who underwent MT, including talc poudrage.^9^ Similar to our patient, Zahra et al. reported a case of bilateral occlusive PE in a patient with active pulmonary TB who was diagnosed with pleural biopsy obtained via VATS. However, the PE incident occurred almost 2 weeks after the thoracoscopy.^18^

In our case, the patient was doing well with no complaints apart from mild pain at the procedure site. However, 2 days after MT, he developed sudden severe respiratory distress and was found to have massive right‐side PE, followed by cardiac arrest and death. Apart from TB, our patient was free from any risk factors for a hypercoagulable state. Furthermore, he was on prophylactic enoxaparin since admission and until 1 day before the procedure. Enoxaparin was not resumed after the procedure as the patient did not require isolation and could ambulate adequately. His Padua prediction score for risk of VTE was less than 4, indicating pharmacologic prophylaxis was not indicated. Although TB itself could be a risk factor for VTE, given the patient was asymptomatic, the close time from the MT, and the absence of other apparent risk factors for VTE, MT had probably served as the predisposing factor for his PE.

In conclusion, although TB can increase the risk of VTE, patients who undergo thoracoscopy may be at a higher risk for developing VTE. This population may require keen observation, appropriate prophylaxis, and imaging to avoid devastating and catastrophic thrombotic complications.

## AUTHOR CONTRIBUTIONS

**Wael Kanjo**: Conceptualization, writing‐original draft. **Shahem Abbarh** and **Amina Bougaila**: Participated in literature review and reviewing the manuscript. **Nagham Sadik** and **Mhd Baraa Habib**: Writing‐review & editing.

## CONFLICT OF INTEREST

None declared.

## FUNDING STATEMENT

Publication fund will be requested from Qatar National Library upon acceptance of the manuscript.

## ETHICS STATEMENT

The authors declare that appropriate written informed consent was obtained for the publication of this manuscript and accompanying images.

## Data Availability

Data sharing is not applicable to this article as no new data were created or analyzed in this study.
